# Experimental and Numerical Studies on Recrystallization Behavior of Single-Crystal Ni-Base Superalloy

**DOI:** 10.3390/ma11071242

**Published:** 2018-07-19

**Authors:** Runnan Wang, Qingyan Xu, Xiufang Gong, Xianglin Su, Baicheng Liu

**Affiliations:** 1 Key Laboratory for Advanced Materials Processing Technology (MOE), School of Materials Science and Engineering, Tsinghua University, Beijing 100084, China; wrn14@mails.tsinghua.edu.cn (R.W.); suxianglin2009@yeah.net (X.S.); liubc@tsinghua.edu.cn (B.L.); 2 State Key Laboratory of Long-Life High Temperature Materials, Deyang 618000, China; gongxf@mail.dfstw.com

**Keywords:** recrystallization, Ni-base superalloy, deformation, simulation

## Abstract

The recrystallization (RX) behavior of superalloy during standard solution heat treatment (SSHT) varies significantly with deformation temperature. Single-crystal (SX) samples of Ni-base superalloy were compressed to 5% plastic deformation at room temperature (RT) and 980 °C, and the deformed samples were then subjected to SSHT process which consists of 1290 °C/1 h, 1300 °C/2 h, and 1315 °C/4 h, air cooling. RT-deformed samples showed almost no RX grains until the annealing temperature was elevated to 1315 °C, while 980 °C-deformed samples showed a large number of RX grains in the initial stage of SSHT. It is inferred that the strengthening effect of γ’ phases and the stacking faults in them increase the driving force of RX for 980 °C-deformed samples. The RX grains nucleate and grow in dendritic arms preferentially when the microstructural inhomogeneity is not completely eliminated by SSHT. A model coupling crystal plasticity finite element method (CPFEM) and cellular automaton (CA) method was proposed to simulate the RX evolution during SSHT. One ({111} <110>) and three ({111} <110>, {100} <110>, {111} <112>) slip modes were assumed to be activated at RT and 980 °C in CPFEM calculations, respectively. The simulation takes the inhomogeneous as-cast dendritic microstructure into consideration. The simulated RX morphology and density conform well to experimental results.

## 1. Introduction

Ni-base superalloys are widely used in industrial gas turbines (IGT) and aero-engines due to their extraordinary mechanical properties and corrosion resistance when servicing in high-temperature and high-pressure conditions [1]. For components with equiaxed crystal structures such as turbine discs, recrystallization (RX) induced by hot-working is treated as an important method to improve the mechanical properties of superalloys. Microstructure refinement during hot working by the process of dynamic RX is a commonly employed approach [2,3]. With respect to single-crystal (SX) components such as turbine blades, new high-angle grain boundaries induced during the RX process can significantly decrease the creep and fatigue resistance [4,5]. Hence, for any aspects, investigation of RX occurrence and microstructure evolution are vital to the application of Ni-base superalloys.

RX behavior in many alloys and their influences on mechanical properties have been widely studied in previous research [6,7,8,9]. Hot mechanical processes such as rolling [10], forging [11], and torsion [12] can lead to grain refinement and corresponding strengthening effects. The RX process, with many controlling parameters such as strain rate, deformation and temperature, is a complicated process. These parameters may interactively influence the degree of solid solution saturation and dislocation density produced during processing, which play the key roles during the RX process [12]. Zhuo et al. [13] reported that formation and migration of dense dislocation walls had been observed in an SX Ni-base superalloy by Transmission electron microscope (TEM); concurrently, the SX matrix was cut into subgrains which are the precursor of cellular RX. In addition, a new RX mode induced by micro-grains of γ’ phases in surface eutectics was observed by Mathur et al. [14]. Recent studies have reported that some second phases such as carbides usually form with the migration of RX grain boundaries due to their high solubility [15].

In order to predict RX process, finite element method (FEM) is widely used to simulate the deformation process and obtain the deformation state [16]. Meanwhile, cellular automaton (CA) [17,18,19] and phase field method [20,21] have been proposed to simulate RX morphology, evolution and density. Reyes et al. [19] used CA method to model the grain size for Inconel 718 superalloy during hot compression at 980 °C and 1020 °C, and the necklace type microstructures were comprehensively predicted. Raabe et al. [22,23] firstly coupled the crystal plasticity finite-element method (CPFEM) with CA method based on the probabilistic analogue of the linearized symmetric rate equation of Turnbull, and successfully simulated the primary static RX in aluminum. Zambaldi et al. [24] simulated the RX formation around the Brinell-type indentation for SX CMSX-4 superalloy, and compared the results to the experiments. The investigations of experiments and simulations have already profoundly enhanced our understanding of microstructure evolution over the past decades, while the studies of RX behavior in the application of standard heat treatment (SSHT) process is rarely reported. Previous studies usually treated the chemical composition of dendritic arms (DAs) and interdendritic regions (IDRs) homogeneously, and used macroscale elastic-plastic constitutive model or one slip system to calculate the deformation behavior of superalloys, neither of which are sufficiently accurate [18,19,24].

In the present study, the influence of deformation temperature and microstructural inhomogeneity on RX nucleating and growing behavior during SSHT were investigated by experiments. Specimens with a single-crystal (SX) structure were employed to investigate the RX formation. A model coupling CPFEM and cellular automaton (CA) was proposed for the simulation of RX evolution during SSHT. Different slip systems were employed for the specimens deformed at different temperature to describe the deformation behavior and calculate the driving force for RX according to reality. Quasi-static compressive tests were conducted at various temperature to validate the CPFEM model. The simulated RX behavior, morphology and grain density in different stages of SSHT were compared with the experiments.

## 2. Materials and Methods

The ϕ 15 mm × 160 mm SX cylindrical bars of DD6 Ni-base superalloy, the nominal chemical composition of which is shown in Table 1, were directionally solidified in an industrial vacuum Bridgman furnace, and spiral grain selectors were added to the very base of them to form SX structure. The orientations of these bars were measured by X-ray Laue method, and only the bars within 12° misorientation angle from [001] were used in the following experiments.

Small ϕ 6 mm × 10 mm cylindrical specimens were cut from the cylinder bars using electrical discharge machining. Quasi-static compressive tests were conducted on a Gleeble-3500D thermo-mechanical simulator (Dynamic Systems Inc., New York and USA). The deformation behavior of superalloy is sensitive to strain rate; in this study the value of strain rate was set as 10^−3^ s^−1^ which can simulate the quasi-static compressive process. The samples were compressed to 40% deformation at various temperatures to obtain strain-stress curves and calibrate CPFEM parameters. Some samples were compressed to 5% plastic deformation and then underwent vacuum SSHT, the process of which includes 1290 °C, 1 h + 1300 °C, 2 h + 1315 °C, 4 h, air cooling, to observe the RX occurrence and morphology. The numbering rule of these specimens is as follows: S—“deformation temperature”—“SSHT number”, the SSHT number is shown in Table 2. For example, S-980-3 represents a specimen which is compressed to 5% plastic deformation at 980 °C and then subjected to part of the SSHT process (1290 °C, 1 h + 1300 °C, 1 h, air cooling).

The annealed cylindrical specimens were cut from the middle section perpendicular to the axial direction and then subjected to mechanical grinding and polishing. The samples were chemical etched using the Marble’s reagent (20 g CuSO_4_ + 100 mL HCl + 100 mL H_2_O) and then observed under optical microscope (OM, Zeiss AM10 OM, Jena, Germany). For the samples with complex microstructure, electron backscattered diffraction (EBSD) detection was conducted after electrochemical polishing in the solution of HClO_4_ (10 mL) and C_2_H_5_OH (90 mL). The Oxford EBSD detector was equipped in a MIRA3 LMH FSEM. The accelerating voltage was 20 kV and the step size was in 10~20 μm range. Raw data was analyzed using HKL CHANNEL5 software to produce inverse pole figure (IPF) and local misorientation map (KAM).

The planimetric procedure in ASTM E112-2013 [25] was employed to determine the average grain density ($\rho_{AG}$) of the specimens. The circle and rectangle counting areas were used to measure the grain number in optical microscope (OM) and electron backscattered diffraction (EBSD) micrographs, respectively. For the rectangle counting area, the corner grain is assumed to have one fourth of its area in the test box, hence the four corner grains together equals one grain within the test box and $\rho_{AG}$ follows: $\rho_{AG}=\frac{N_{Inside}\;+\;0.5N_{Intercepted}\;+\;1}{A}$, where *A* is the test area in mm^2^, *N_Inside_* is the number of grains completely inside the test rectangle and *N_Intercepted_* is the number of grains that intercept the test rectangle. For the circle counting area, $\rho_{AG}=\frac{N_{Inside}\;+\;0.5N_{Intercepted}}{A}$. Commercial thermodynamic calculating software, JMatPro6.1 and Nickel Based Superalloy database, were employed to determine the fraction of γ’ phase in DAs and IDRs based on the local compositions quantitatively determined by Electron probe micro-analyzer (EPMA) [26].

## 3. Mathematical Model

### 3.1. Crystal Plasticity Finite-Element Method

Compared to the conventional elastic-plastic constitutive model, crystal plasticity finite-element method (CPFEM) based on slip theory has gained rapid development in the last two decades. It gradually became an extremely versatile tool for describing the mechanical response of crystalline materials on all length scales from single crystals to engineering parts [27].

In crystal plasticity theory there are two basic kinematics assumptions: (1) the plastic deformation only arises from the slip of dislocation alone the certain direction on the activated slip plane; (2) the elastic strain leads to the stretching and rotation of lattice. The total deformation gradient **F** is decomposed into two parts containing plastic shear ($F^{p}$) and elastic deformation ($F^{e}$) through:

(1) $$F=F^{e}F^{p}$$

The velocity gradient **L** in current configuration follows:

(2) $$L=\dot{F}F^{-1}=D+\Omega ={\dot{F}}^{e}F^{e^{-1}}+{\dot{F}}^{e}{\dot{F}}^{p}F^{p^{-1}}F^{e^{-1}}$$

where the symmetric rate of stretching (**D**) and the antisymmetric spin tensor (**Ω**) can be decomposed into lattice parts (**D***^e^* and **Ω***^e^*) and plastic parts (**D***^p^* and **Ω***^p^*) as: $D=D^{e}+D^{p}$, $\Omega =\Omega^{e}+\Omega^{p}$, $D^{e}+\Omega^{e}={\dot{F}}^{e}F^{e-1}$ and $D^{p}+\Omega^{p}=\sum_{\alpha }{\dot{\gamma }}^{\alpha }s^{e\left(\alpha \right)}m^{e\left(\alpha \right)}$. The vector of slip direction ($s^{e\left(\alpha \right)}$) and the normal to the slip plane ($m^{e\left(\alpha \right)}$) in the deformed configuration follows: $s^{e\left(\alpha \right)}=F^{e}s_{0}^{\alpha }$ and $m^{e\left(\alpha \right)}=m_{0}^{\alpha }F^{e-1}$ respectively, where $s_{0}^{\alpha }$ and $m_{0}^{\alpha }$ are the vectors in the reference configuration.

The rate of change of $F^{p}$ follows: ${\dot{F}}^{p}F^{p^{-1}}=\sum_{\alpha }{\dot{\gamma }}^{\alpha }s_{0}^{\alpha }⊗m_{0}^{\alpha }$, where ${\dot{\gamma }}^{a}$ and $s_{0}^{\alpha }⊗m_{0}^{\alpha }$ denote the slipping rate and the Schmid matrix, respectively. The relationship between the Jaumann rate ${\overset{\nabla }{\sigma }}^{e}$ and Cauchy stress **σ** follows:

(3) $${\overset{\nabla }{\sigma }}^{e}+\sigma \left(I:D\right)=L:D^{e}$$

(4) $${\overset{\nabla }{\sigma }}^{e}=\overset{\nabla }{\sigma }+\left(\Omega -\Omega^{e}\right)\cdot \sigma -\sigma \cdot \left(\Omega -\Omega^{e}\right)$$

(5) $$\overset{\nabla }{\sigma }=\dot{\sigma }-\Omega \cdot \sigma +\sigma \cdot \Omega$$

where **I** is the second-order unit tensor and $\overset{\nabla }{\sigma }$ the co-rotational stress rate on the axes spinning with material. The crystalline slip depends on **σ** through the Schmid stress $\tau^{\alpha }=m^{e\left(\alpha \right)}\cdot \frac{\rho_{0}}{\rho }\sigma \cdot s^{e\left(\alpha \right)}$, where *ρ_0_* and *ρ* are the reference and current mass density. Rate of change of Schmid stress can be written as: ${\dot{\tau }}^{\alpha }=m^{e\left(\alpha \right)}\cdot \left[\overset{\nabla }{\sigma }+\sigma \left(I:D^{e}\right)-D^{e}\cdot \sigma +\sigma \cdot D^{e}\right]\cdot s^{e\left(\alpha \right)}$.

Rate-dependent hardening law was employed in this model: ${\dot{\gamma }}^{\alpha }={\dot{\gamma }}_{0}\mathrm{sgn}\left(\tau^{\alpha }\right){\left|\frac{\tau^{\alpha }}{g^{\alpha }}\right|}^{m}$, where ${\dot{\gamma }}_{0}$ and $g^{\alpha }$ are reference shearing rate and slip resistance of the α slip system. The rate of change of *g^α^* follows: ${\dot{g}}^{\alpha }=\sum_{\beta }h_{\alpha \beta }{\dot{\gamma }}^{\beta }$, where $h_{\alpha \beta }$ is the hardening modulus and ${\dot{\gamma }}_{\beta }$ the shearing rate of *β* slip system (self-hardening: *α* = *β*; latent-hardening: *α* ≠ *β*). $h_{\alpha \beta }=qh_{0}{\mathrm{sech}}^{2}\left|\frac{h_{0}\gamma }{\tau_{s}-\tau_{0}}\right|$, where *h_0_* is the initial hardening modulus, $\tau_{0}$ the yield stress, $\tau_{s}$ the saturated stress and *q* the constant. For latent-hardening, $\gamma =\sum_{\alpha }\int_{0}^{t}\left|{\dot{\gamma }}^{\alpha }\right|dt$, and *q* is chosen in range of 1–1.4 [28].

The driving force for RX nucleating and growing is the deformation-stored energy (*E_store_*) induced by plastic strain. The Hirsch dislocation forest theory relates the strength of α slip system (*g^α^*) and the dislocation density ($\rho_{\alpha }^{D}$) by Bailey–Hirsch relation [29,30,31]:

(6) $$g^{\alpha }=g^{0}+c\mu b\sqrt{\rho_{\alpha }^{D}}$$

(7) $$E_{store}=\frac{1}{2}\sum_{\alpha }\rho_{\alpha }^{D}\mu b^{2}$$

where *g^α^* and *g^0^* are deformed and initial strength of the α slip system, *μ* the shear modulus (derived from the tensile test, RT: 142 GPa; 980 °C: 85.2 GPa), ***b*** the Burger’s vector and *c* the constant of the order of unity 0.1.

The simulation parameters were obtained by calibration using quasi-static compressive tests, and compared with the parameters used in other superalloy of same generation such as CMSX-4 [24,32]. The slip systems activated at various temperature and the parameters used in the CPFEM simulation are presented in Section 4.1.

### 3.2. CA Model for Recrystallization Simulation

The CA model consists of RX nucleating, growing and coarsening. The driving force for RX formation is the plastic strain, which is essentially the dislocation induced during deformation process. In this simulation, it is derived from the results of CPFEM calculation. The RX nucleation is related to the migration of dislocation walls and the coalescence of subgrains, but CA simulation approach works above this scale. A phenomenological nucleation model considering the kinetic and thermodynamic instability criterion was used. The driving force (*P*) for RX nucleation is the stored energy calculated from the dislocation density (Equation (7)). The RX nucleus usually occurred in the region with large deformation, so the continuous nucleation model is suitable for describing this process [24,33]. The nucleation rate $\dot{N}$ can be expressed as:

(8) $$\dot{N}=C_{0}\left(P-P^{c}\right)\exp\left(-\frac{Q_{a}}{RT}\right)$$

where *C_0_* is constant coefficient (1.0 × 10^9^ s^−1^·J^−1^), *R* the Molar gas constant (8.3144 J·mol^−1^·k^−1^), *T* the temperature (in degree Kelvin), *P* the driving force (*E_store_*) and *Q_a_* the activation energy for RX nucleation. *P^c^* is the critical stored energy for RX nucleation following $p^{c}=\frac{10^{7}\varepsilon_{c}}{2.2\varepsilon_{c}\;+\;1.1}E_{low}$, where *ε_c_* and *E_low_* denote the critical plasticity and low-angle grain-boundary energy, respectively. The critical temperature for RX nucleation was determined according to the experimental results of isothermal heat treatment in Reference [34], and those of S-RT and S-980 with 5% plastic deformation are 1310 °C and 1260 °C, respectively.

The velocity of grain boundary migration *V* follows:

(9) V=MP

where *M* is the grain boundary mobility for the static recrystallization, *P* the driving pressure for the grain boundary movement. *M* follows the Arrhenius formula (Equation (10)), in which *M_0_* is the constant of grain boundary mobility, *Q_b_* the activation energy for grain boundary motion, *D_0_* the diffusion constant and *k* the Boltzmann constant.

(10) $$M=M_{0}\exp\left(-\frac{Q_{b}}{RT}\right)=\frac{D_{0}b^{2}}{kT}\exp\left(-\frac{Q_{b}}{RT}\right)$$

After the RX process is completed, the driving force (*P*) for grain coarsening is the grain boundary energy *E* (Equation (12)) and curvature *κ* (Equation (13)), which follows Equation (11).

(11) P=Eκ

(12) $$E=E_{m}\left(\frac{\theta }{\theta_{m}}\right)\left(1-\ln\left(\frac{\theta }{\theta_{m}}\right)\right)$$

(13) $$\kappa =\frac{a}{c_{s}}\frac{N_{0}-c_{i}}{N^{\prime }}$$

where *E_m_* is high angle grain boundary energy, *θ* the misorientation angle of adjacent grains, *θ_m_* the critical misorientation for high angle grain boundary (15°), *a* the constant (1.28), *c_s_* the CA element size, *N_0_* the element number needed for planar interface (15), *N’* the number of Moore cells (25) and *c_i_* the number of elements having the same state as center element.

The CA capturing is implemented in the Moore-type neighboring elements which take the cells of the neighboring two layers into consideration. The value of *Q_a_* and *Q_b_* are different in DAs and IDRs because the microsegregation induced during solidification can lead to a different solution behavior of secondary phase γ’, which act as hindering sites during the migration of RX grain boundaries in these two regions. Key parameters employed are shown in Table 3. Dendritic microstructure in different stage of SSHT was derived from the image processing technology by changing the dendritic regions artificially based on experimental observation.

## 4. Results and Discussion

### 4.1. Temperature Dependence of Slip System in the Superalloys

**Figure 1:**
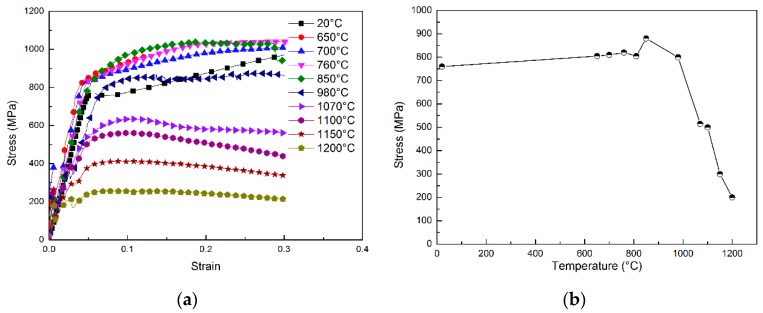
Deformation behavior of [001] orientated single-crystal samples: (**a**) stress-strain curves for the quasi-static compression at different temperature; (**b**) corresponding yield stress curve.

The true strain-true stress curves for the samples undergoing quasi-static compressive tests at various deformation temperature are shown in Figure 1a. A curved shape conforming to a classic elastic-plastic constitutive relationship can be observed, which shows a linear elastic relationship and a clear yield behavior on the two sides of the yield point. The corresponding yield stress at different temperatures is given in Figure 1b, from which it can be concluded that the Ni-base superalloy continually hardens with the increase in temperature until approximately 850 °C, and then softens when the temperature exceeds 1000 °C.

**Figure 2:**
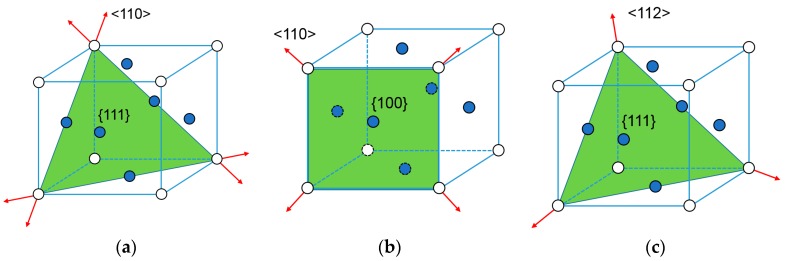
Schematic illustration of activated slip system in face-centered cubic metals at various temperature: (**a**) <110> {111}; (**b**) <110> {001}; (**c**) <112> {111}.

The temperature dependence of deformation mechanisms in SX Ni-base alloys has been investigated through experiments [28,35,36]. Plastic deformation at ambient temperature occurs in face-centered cubic (FCC) metals mainly through the activated slip system consisting of <110> and {111}. When temperature increases to 800 °C, slip-line analysis in a deformed SX superalloy has revealed that the mode of slip deformation changes from α/2 <110> {111} to α/2 <110> {001} [1]. Reference [37] indicates that the slip modes activated at different temperature can be divided into three types for DD3 superalloy: (1) <110> {111} below 600 °C; (2) <110> {111} and <110> {001} in the range of 600 °C~850 °C; (3) <110> {111}, <110> {001} and <112> {111} above 850 °C. These three slip modes, octahedral (Oct1) <110> {111}, hexahedral (Cub) <110> {100} and dodecahedral (Oct2) <112> {111} have 12, six and 12 individual slip systems, respectively [38]. At 980 °C, three types of slip modes (<110> {111}, <110> {100}, <112> {111}) are assumed to be activated in DD6 Ni-base superalloy according to Reference [38]. The slip mode activated at different temperatures for DD6 superalloy is presented in Table 4, and corresponding schematic illustration of activated slip systems are shown in Figure 2.

The variations of activated slip systems are explained by the variation of dislocation Peierls–Nabarro stress on crystal planes at different temperature [39]. The crystal plane with minimum Peierls-Nabarro stress at high temperature is different from that at low temperature. Another opinion is that the slip on the sub close-packed lattice plane of FCC crystal (001) is controlled by the cross slip of screw dislocation, which is essentially a process of thermal activation. Hence, the slip of the sub close-packed lattice plane tends to occur at high temperature. Various dislocation movement mechanisms were proposed to describe the deformation behavior of superalloy, such as antiphase boundary (APB) shearing, stacking fault (SF) shearing and Orowan bypassing [29,40,41].

**Figure 3:**
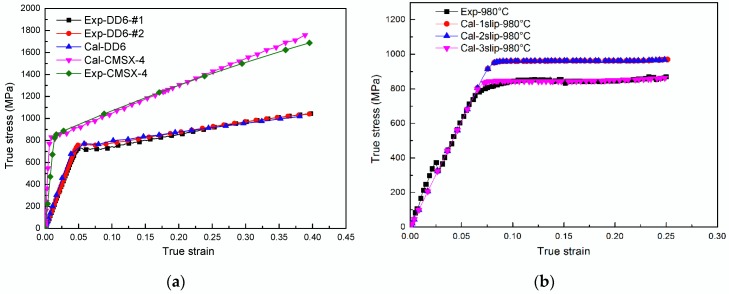
Stress-strain curves for quasi-static compressive test of [001] oriented SX samples. (**a**) DD6 and CMSX-4 superalloy deformed at room temperature with one slip mode activated; (**b**) DD6 deformed at 980 °C, with various slip modes activated.

The calculated (Cal) and experimental (Exp) stress-strain curves for CMSX-4 superalloy and DD6 superalloy deformed at RT are shown in Figure 3a. The parameters of CMSX-4 superalloy are from Reference [24], and those of DD6 superalloy are calibrated from quasi-static compressive test. The parameters used for CPFEM calculation are presented in Table 5. The simulated results of RT-deformation agree well with the experimental results of Reference [24] and the present compression experiments, as shown in Figure 3a. The hardening parameters are assumed to be identical for different slip systems at elevated temperature owing to the similar underlying characteristic dislocation reactions [42,43]. The CPFEM calculation with one, two and three slip modes activated were tested at 980 °C as shown in Figure 3b. This indicates that the slip modes which are assumed to be activated can significantly influence calculation results. Actually, for the samples compressed along [001] orientation, the Schmid factors of all slip systems of <110> {001} slip mode μ=cosλcosϕ=0, hence this slip mode is not activated in present test condition. This explains why the simulated strain-stress curves with one and two slip modes activated overlap with each other.

### 4.2. Microstructural Evolution

**Figure 4:**
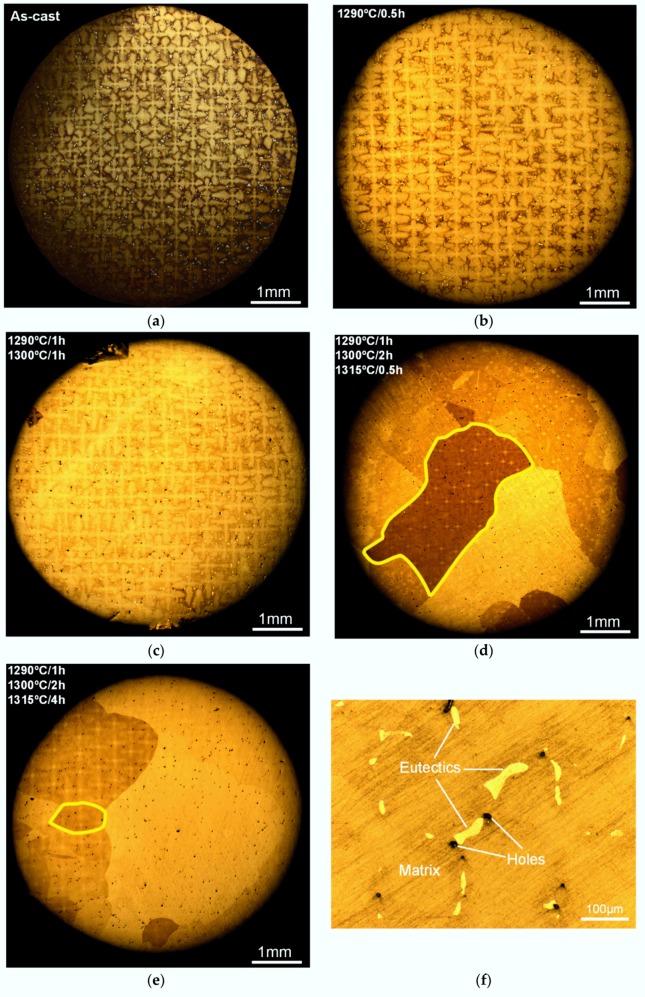
Microstructural evolution of S-RT during SSHT procedure. (**a**) As-cast deformed; (**b**) S-RT-2; (**c**) S-RT-3; (**d**) S-RT-4; (**e**) S-RT-5; (**f**) holes near eutectics.

The microstructural evolutions of S-RT (specimens deformed at RT) in different stages of SSHT under OM are shown in Figure 4. Some newly formed RX grains are marked by the yellow line. With the increase in annealing temperature and holding time, the elemental microsegregation between DAs and IDRs gradually decreases, as well as the area of IDRs and γ/γ’ eutectics, leading to a progressively homogeneous SX matrix. In an as-cast deformed sample (Figure 4a), dendritic morphology can be easily identified from the OM micrograph owing to the different solidification sequence of DAs and IDRs and elemental microsegregation. Almost no RX grain forms in the first (1290 °C, Figure 4b) and second (1300 °C, Figure 4c) stages of SSHT. In the third stage, irregular RX grains rapidly grow until the whole matrix is occupied (Figure 4d,e). The eutectics dissolved during heat treatment and the holes formed near them (Figure 4f). In OM micrograph these holes seem like black spots (Figure 4e,f).

**Figure 5:**
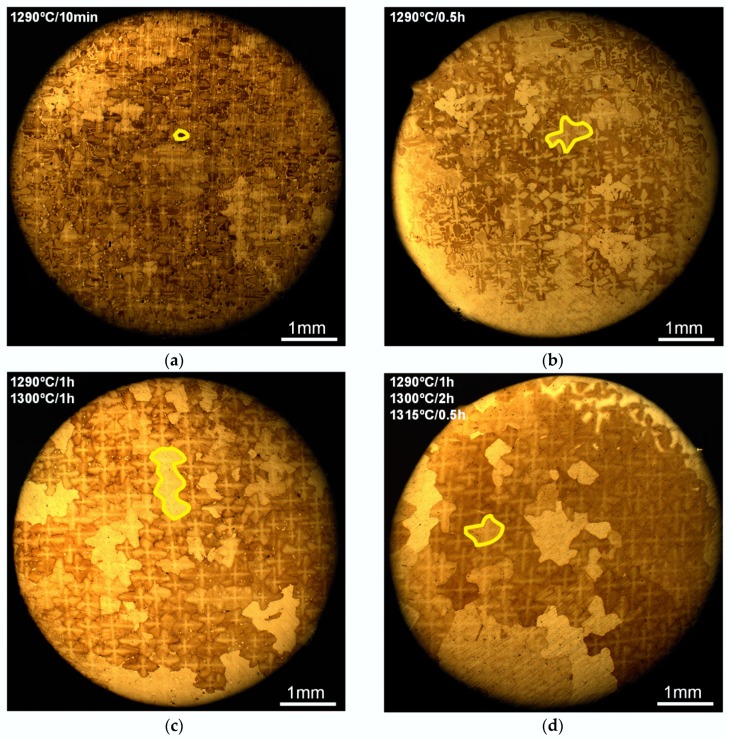

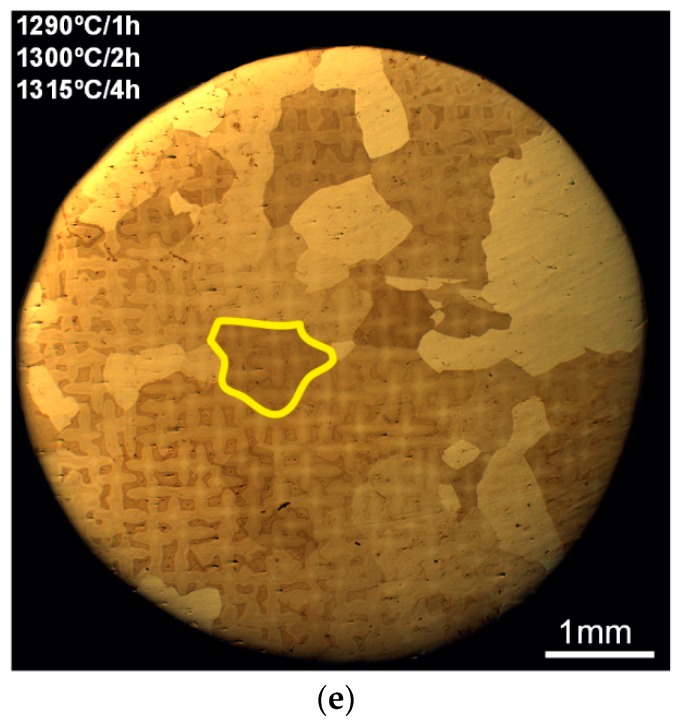
Microstructural evolution of S-980 during SSHT procedure. (**a**) S-980-1; (**b**) S-980-2; (**c**) S-980-3; (**d**) S-980-4; (**e**) S-980-5.

The microstructural evolutions of S-980 (specimens deformed at 980 °C) are shown in Figure 5. As shown in Figure 5a, many RX nuclei appear in the initial period (10 min) of the first stage of SSHT process, which is quite different from the observation of S-RT. This indicates that setting different critical temperatures for RX nucleating according to deformation temperature is reasonable. The RX grains show dendritic morphology over a long period of SSHT (Figure 5b,c). With the increase of annealing temperature and holding time, the number of RX grains significantly decreases, and the grains gradually evolve to an irregular morphology (Figure 5e).

The samples deformed at different temperature show different RX behavior during the SSHT procedure. Cox [44] and Panwisawas [16] drew the conclusion that RX grains tend to occur in the samples deformed at elevated temperatures, while Li [34] found the highest RX propensity occurs in the samples deformed at 980 °C for DD6 superalloy. From TEM observation it is inferred that a large amount of stacking faults induced at 980 °C could facilitate RX through thermal twinning nucleation [34]. This could be an important factor in the improvement of RX propensity. Besides, the strengthening effect of γ’ phases increases the superalloy strength at elevated temperature, and the γ’ phases play a role of second phase particles which can lead to an increase of dislocation density in the deformed matrix by several orders of magnitude as dislocations pass around the particles. Hence the equivalent strain induced at elevated temperatures can produce a higher dislocation density and stored energy compared to RT, which conforms with the TEM observation of Cox’s [44] and Li’s [34] studies. The simulation results present the same tendency, that the calculated stored energy of S-RT and S-980 are 1.56 × 10^6^ J/m^3^ and 2.75 × 10^6^ J/m^3^, respectively. With the increase of alloy strength and the number of slip systems at elevated temperatures, the dislocation density and corresponding energy stored in the deformed samples increases as well. 

**Figure 6:**
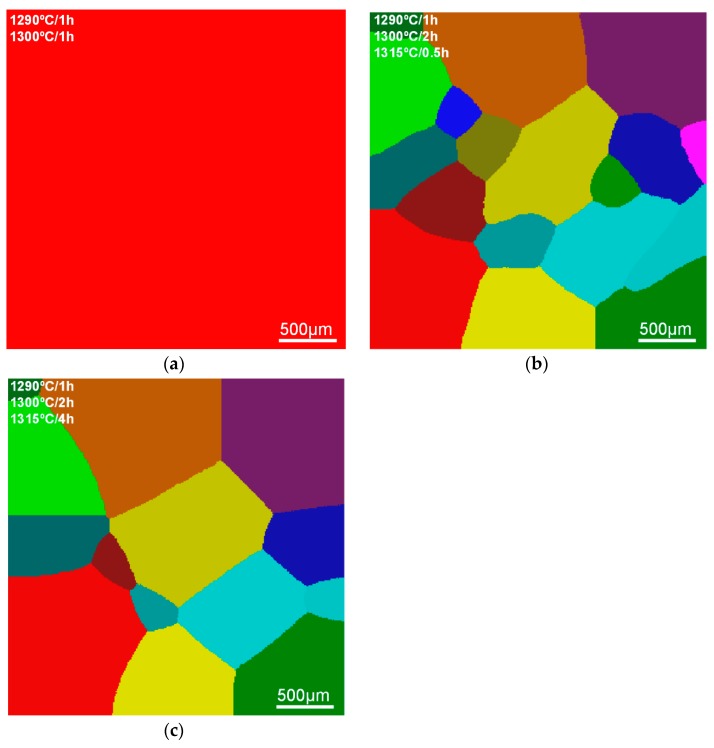
Simulated RX morphologies of S-RT during SSHT. (**a**) S-RT-3; (**b**) S-RT-4; (**c**) S-RT-5.

**Figure 7:**
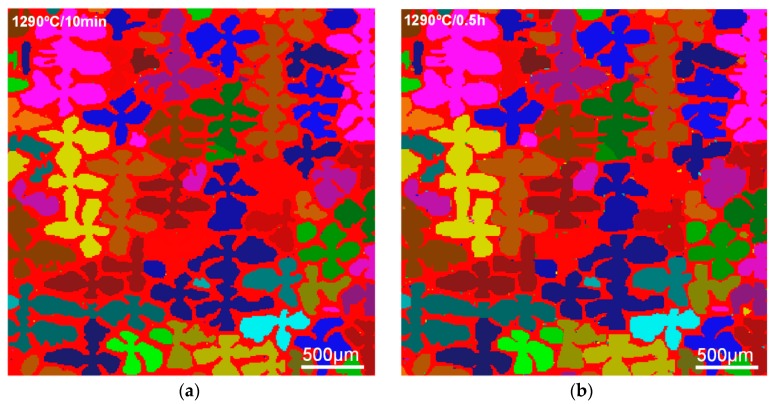

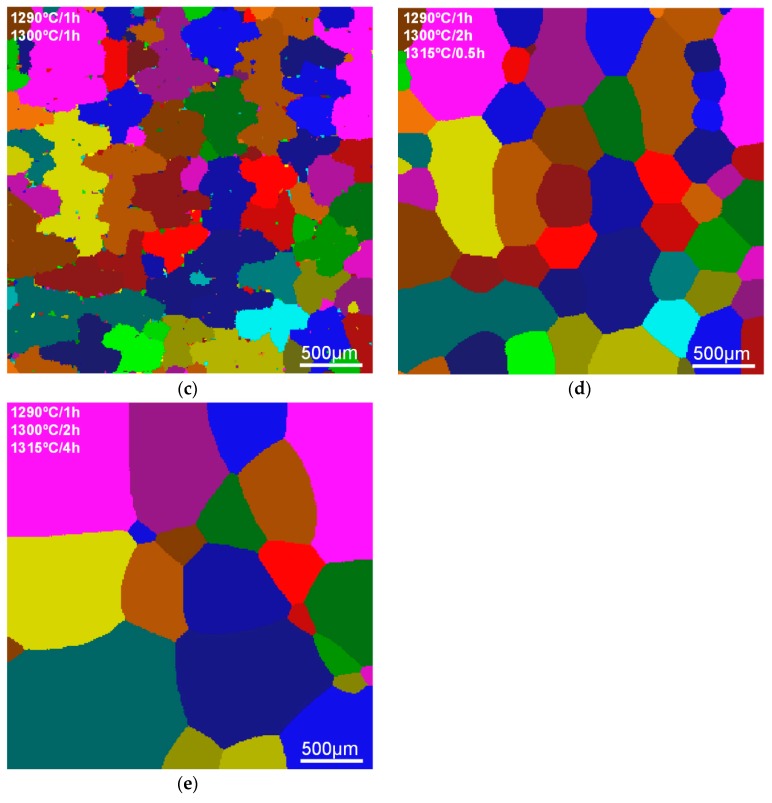
Simulated RX morphologies of S-980 during SSHT. (**a**) S-980-1; (**b**) S-980-2; (**c**) S-980-3; (**d**) S-980-4; (**e**) S-980-5.

The simulated RX morphologies of S-RT and S-980 are shown in Figure 6 and Figure 7, respectively. As the critical nucleation temperature for S-RT reaches as high as 1310 °C, no RX grains appear in the first (1290 °C) and second (1300 °C) stage of SSHT process (Figure 6a). In the third stage (1315 °C), the γ’ phases have completely dissolved into the matrix, and the whole region has become homogeneous. Hence, the RX can nucleate and grow homogeneously (Figure 6b,c), which is quite different from S-980. For S-980, RX initially nucleates in DAs and grows individually with a dendritic morphology (Figure 7a). Migration of RX grain from DAs to IDRs gradually proceeds until they contact each other (Figure 7c), and then the grains continuously coarsen and compete until the end of SSHT.

### 4.3. Dependence of Inhomogeneous Microstructure on RX Behavior

**Figure 8:**
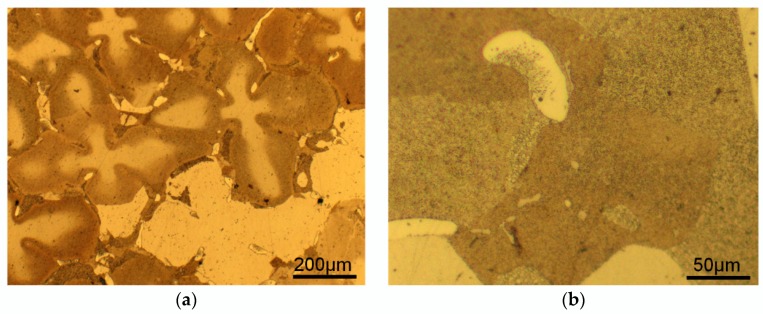
Influence of microstructural heterogeneities on the RX behavior of S-980-2. (**a**) DA and IDRs; (**b**) eutectic particle.

A higher magnification of some regions in Figure 5b is shown in Figure 8. For S-980, most of the RX grains arise from the DAs and grow individually (Figure 8a). The migrations of RX grain boundaries stop outside the eutectic particles (Figure 8b). According to previous research [45,46,47,48], RX is very sensitive to secondary phase particles or microstructural inhomogeneity, while undissolved γ’ phase and incoherent eutectic particles act exactly as these kinds of barriers to retard the migration of RX grain boundaries owing to various pinning mechanisms.

**Figure 9:**
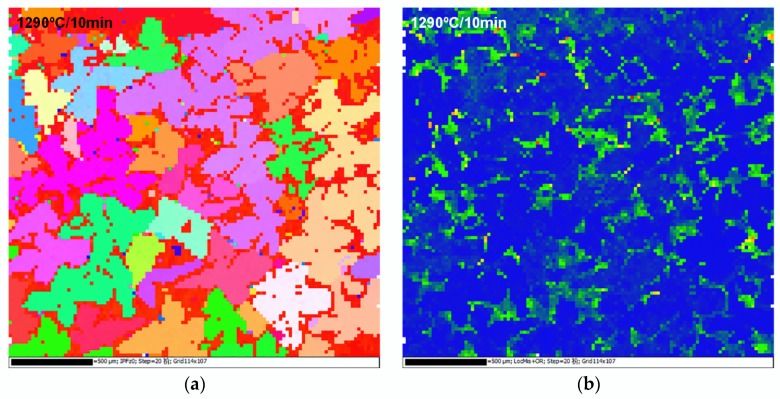

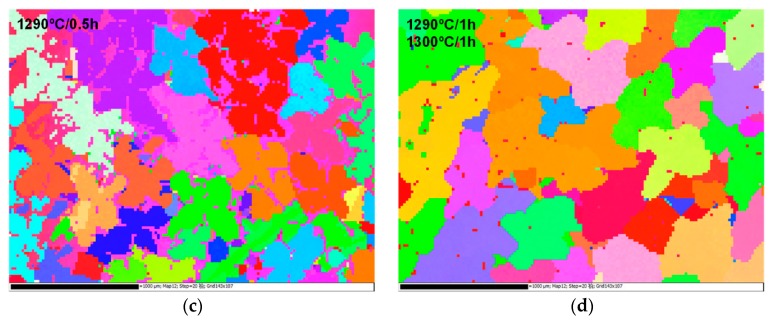
Electron backscattered diffraction (EBSD) results of S-980: (**a**) S-980-1 inverse pole figure (IPF); (**b**) S-980-1 local misorientation map (KAM); (**c**) S-980-2 IPF (**d**) S-980-3 IPF.

The microstructure of S-980-1, S-980-2 and S-980-3 are hard to distinguish in the OM micrographs; hence EBSD technology is employed to test these specimens. The IPF maps of S-980-1, S-980-2 and S-980-3 are shown in Figure 9a, c and d, and the KAM map of S-980-1 is shown in Figure 9b. The microstructure evolution in IPF maps strongly indicate that RX firstly nucleates and grows in DAs, while most IDRs still maintain SX state (Figure 9a,c). The local misorientation in recrystallized areas (DAs) tends to be close to zero, while that of unrecrystallized IDRs is higher, revealing that the deformation matrix still exists in IDRs (Figure 9b). With the increase of annealing temperature and holding time, the recrystallized areas were gradually enlarged and migrated from DAs to IDRs until they overgrew the whole deformed matrix (Figure 9d). As the secondary phase particles, the eutectics are hard to dissolve even after SSHT, and they still exist in the recrystallized areas (red spots in Figure 9d) when the RX process completes.

The DD6 Ni-base superalloy consists of ten elements, and they can exacerbate the microstructural and chemical heterogeneities. The elements enriched in DAs are Co, W and Re, while those enriched in IDRs are Al, Cr, Mo, Ta and Nb [26]. As the main precipitated strengthening phases, γ’ phase plays a role in deformation mechanism of Ni-base superalloys. Dislocations can accumulate around the cubic-shaped γ’ phase. In addition, the migration of RX grain boundaries is a process controlled by solute redistribution and diffusion; hence, the solution behavior of the extant phases significantly influences the RX behavior. The elemental compositions in γ, γ’ phases, DAs, IDRs and γ/γ’ eutectics are different, and hence the RX behavior depends on regions.

**Figure 10:**
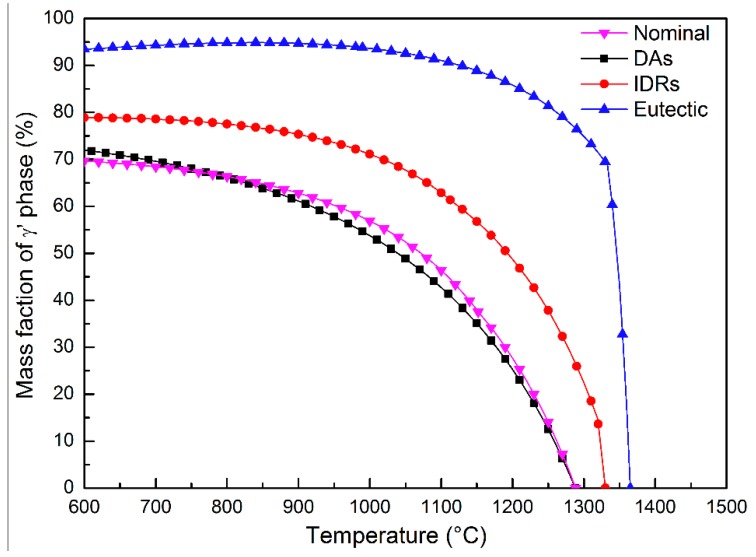
Solution behavior of γ’ phase in DAs, IDRs and eutectics obtained from thermodynamic calculation.

Based on the chemical compositions quantitatively detected by EPMA (Table 6), which are reported in our previous study [26], the solution behavior of γ’ phases in DAs, IDRs and eutectics is calculated by the commercial thermodynamic calculating software JMatPro. As shown in Figure 10, the full solution temperatures of DAs, IDRs and γ/γ’ eutectics are 1284 °C, 1330 °C and 1364 °C, respectively. This means that the region will become homogeneous when it reaches this temperature. Although the calculation is conducted based on the database measured from experiment, this value may not absolutely conform with the solidification reality, but the tendency still has important reference meaning. The full solution temperatures of the γ’ phase in different regions follow the order of Das < nominal composition < IDRs <γ/γ’ eutectics, which conforms with the solution behavior of γ’ phase reported in Reference [49]. Because the critical RX nucleating temperature of S-RT are higher, the influence of solution sequence on RX behavior cannot be observed in them. However, for S-980, the γ’ phases mostly dissolved into a γ matrix in the first stage (1290 °C) of SSHT, while the γ’ phases in IDRs only dissolved by 50% according to the thermodynamic calculation. Hence, each dendritic arm became an individual homogeneous region. Once RX nucleation occurred, they could rapidly grow in such regions. With the increase of annealing temperature and holding time, the γ’ phases in IDRs gradually dissolved, and the RX grain boundaries migrated from DAs to IDRs. However, the dissolution temperature of γ’ phases in eutectics is the highest and the dissolution rate is very slow; hence, these regions are most difficult for RX grain to occupy, and the SX eutectics can still be observed in Figure 9d (red spots).

### 4.4. Variation of Grain Density

**Figure 11:**
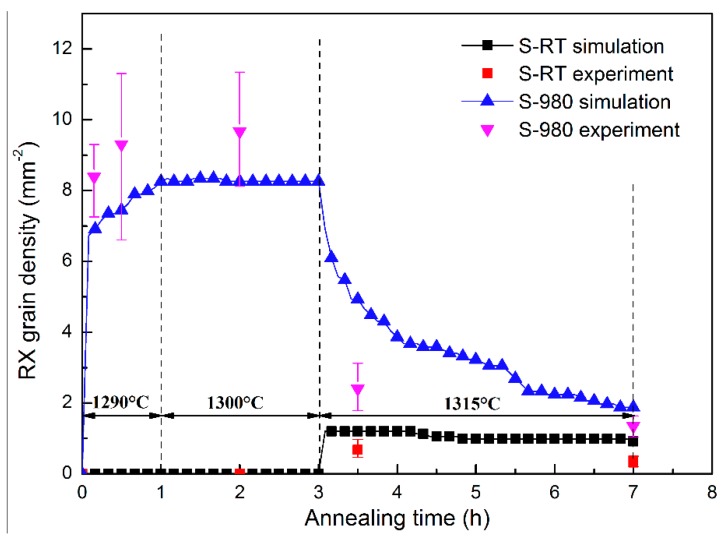
Variation of simulated and experimental grain density with annealing temperature and holding time during SSHT process.

The variation of experimental and simulated RX grain densities in different stages of SSHT are shown in Figure 11. A grain density of S-980 is larger than S-RT through the whole SSHT process. The peak grain density of S-980 reaches 9 mm^−2^ in the first two stages of SSHT, while that of S-RT only reaches 1 mm^−2^ in the third stage of SSHT. This should be ascribed to the stored energy and matrix structure. The calculated stored energy in S-980 is almost twice that in S-RT. More stored energy facilitates the nucleation process during SSHT. In the initial stage of SSHT, many nuclei appear in individual DAs which are separated by IDRs for S-980. This retards the migration of RX grain from recrystallized areas to unrecrystallized areas; hence, the unrecrystallized areas have more opportunity to nucleate, whereas the RX nuclei of S-RT occur in the third stage of SSHT, in which the whole matrix has become homogeneous, and RX grains can rapidly grow until collision. When the RX process completes, grains gradually coarsen under the control of grain boundary energy and curvature, resulting in a significant reduction in grain density.

## 5. Conclusions

The influence of deformation temperature on RX behavior during the SSHT process was investigated in SX Ni-base superalloy. A model coupling CPFEM and CA method was proposed to simulate the deformation process and RX evolution. The following conclusions can be drawn:

1. During the deformation process of DD6 SX superalloy, with the increase of deformation temperature, the alloy strength increases before 850 °C and then decreases. Moreover, the number of activated slip modes increases from one to three. The simulated strain-stress curves of CPFEM agree well with the quasi-static compression tests;
2. The occurrence of RX is quite different in the samples deformed at different temperatures. RX nuclei appear in the initial stage of SSHT for S-980 and the RX grains retain a dendritic morphology. For S-RT, RX does not occur until the third stage of SSHT, and the RX grains grow rapidly with an irregular morphology after nucleating;
3. The RX behavior strongly depends on the microstructural inhomogeneity, due to the difference of solution behavior of γ’ phases in different regions. RX tends to initially nucleate and grow in DAs. With the increase in homogeneity of DAs and IDRs, the RX grains migrate from DAs to IDRs and contact each other, but still retain a dendritic morphology. When the RX process completes, the grains continuously coarsen under the control of grain boundary energy and curvature, and the grain density rapidly decreases;
4. The model coupling CPFEM and CA is capable of describing the RX evolution process during SSHT. The simulated RX behavior, morphology and grain density agree with the experimental results. The grain density of S-980 is higher than S-RT during the whole SSHT process; the former peak value reaches 9 mm^−2^, while the latter only reaches 1 mm^−2^.

## Figures and Tables

**Table 1:** Nominal chemical composition of DD6 superalloy.

Element	Cr	Co	Mo	W	Ta	Re	Nb	Al	Hf	Ni
wt %	4.3	9	2	8	7.5	2	0.5	5.6	0.1	Balance

**Table 2:** Standard solution heat treatment (SSHT) number applied in presented investigation.

SSHT Number	Stage of SSHT Process
1	1290 °C, 10 min, air cooling
2	1290 °C, 30 min, air cooling
3	1290 °C, 1 h + 1300 °C, 1 h, air cooling
4	1290 °C, 1 h + 1300 °C, 2 h + 1315 °C, 0.5 h, air cooling
5	1290 °C, 1 h + 1300 °C, 2 h + 1315 °C, 4 h, air cooling

**Table 3:** Key parameters used in cellular automaton (CA) simulation.

Parameter	Value	Unit
Activation energy for nucleation in dendritic arms, *Q_a1_*	285~305	kJ∙mol^−1^
Activation energy for nucleation in interdendritic regions, *Q_a2_*	290~310	kJ∙mol^−1^
Activation energy for recrystallization in dendritic arms, *Q_b1_*	250~265	kJ∙mol^−1^
Activation energy for recrystallization in interdendritic regions, *Q_b2_*	335~350	kJ∙mol^−1^
Time step	0.01	s
Burgers vector, *b*	0.36	nm
Cell size	0.005	mm

**Table 4:** Slip systems activated at different temperature for DD6 superalloy.

Temperature	Activated Slip Modes		
<600 °C	<110> {111}		
600 °C–810 °C	<110> {111}	<110> {001}	
>810°C	<110> {111}	<110> {001}	<112> {111}

**Table 5:** Parameters used in crystal plasticity finite element method (CPFEM) simulations.

Parameter		CMSX-4 (RT) [24]	DD6 (RT)	DD6 (980 °C)	Unit
Elastic moduli,	*C_11_*	252	25.6	20.8	GPa
	*C_12_*	161	12.1	12.1	GPa
	*C_44_*	131	14.9	14.9	GPa
Reference strain rate,	${\dot{\gamma }}_{0}$	0.001	0.001	0.001	s^−1^
Initial slip resistance	$\tau_{0}$	245	320	403	MPa
Saturation slip resistance	$\tau_{s}$	775	580	450	MPa
Power law exponent	*m*	20	20	20	1
Initial hardening modulus	*h_0_*	350	145	93	MPa
Hardening ratio	*q*	1	1	1~1.4	1

**Table 6:** Average chemical composition (in wt %) of DAs, IDRs and γ/γ’ eutectics [26].

Region	Ni	Al	Cr	Co	W	Mo	Ta	Nb	Re	Hf
DA	65.069	6.114	3.838	9.231	8.351	1.41	4.611	0.381	2.446	-
IDR	64.075	6.467	4.070	8.300	4.401	1.966	7.827	1.562	1.725	0.131
Eutectic	67.501	7.610	1.953	6.794	3.238	0.852	10.830	1.340	0.445	0.012
